# Behind the Shirt: Examining Trends in the Commercial Relationships Between English Premier League Football Clubs and Gambling Companies

**DOI:** 10.12688/wellcomeopenres.27602.1

**Published:** 2026-10-06

**Authors:** Fabiola Creed, Heather Wardle

**Affiliations:** 1 University of Glasgow School of Social and Political Sciences, Glasgow, Scotland, UK

**Keywords:** gambling, sport sponsorship, football, marketing, fan activations, front-of-shirt, history, policy

## Abstract

**Background:**

In August 2026, the English Premier League (EPL) banned front-of-shirt gambling sponsorships to reduce gambling marketing exposure. Shirt sponsorship represents one aspect of broader commercial relationships between football clubs and gambling companies, often not covered by existing industry codes. We charted historical commercial arrangements between gambling companies and leading EPL clubs that have never had front-of-shirt gambling sponsorships, examining marketing practices and commercial partnerships that will remain after the ban.

**Methods:**

The study analyses the presence of gambling partnerships with the five top-performing EPL clubs from 2000/01 to 2025/26 (Manchester United, Arsenal, Chelsea, Liverpool, Manchester City), collecting data from club websites, digital archives, match footage, and press sources. Within each partnership, we coded up to 13 marketing features across four themes: clothing, stadia, digital integration, and fan activations.

**Results:**

None of the clubs had front-of-shirt gambling sponsors, yet across 25 years they formed 91 distinct partnerships with gambling companies. The total number of commercial gambling partnerships increased over time, with clubs entering multiple partnerships in a single season during an era of expanding digital marketing and direct consumer engagement. Fan-facing activations as a feature of commercial arrangements have substantially increased over time. Offering fans exclusive odds and competitions/experiences was present in over 80% of the commercial partnerships reviewed. Other common features included co-branded digital content, which utilises social media and first-team players (82.4%), and pitch-side advertising (71.4%).

**Conclusions:**

The ban on front-of-shirt sponsorship addresses only one element of football’s relationship with the gambling industry. EPL clubs engage in a wide range of gambling-related commercial activities, particularly through fan activations, that fall beyond the remit of existing voluntary Codes of Conduct for Gambling Related Agreements in football. These marketing channels will remain available after the ban, indicating that substantial levels of fan exposure to gambling marketing are likely to persist.

## Introduction

Gambling marketing, advertising and sponsorship have a recognised impact on gambling behaviours, with systematic review evidence showing a dose-response relationship between greater exposure to gambling advertising and increased gambling consumption. ^1^ ^,^ ^2^ Partnerships with and sponsorship of professional sporting teams by gambling companies have increased vastly over the last 25 years. ^3^ ^,^ ^4^ In the 2025/26 English Premier League season (EPL), 11 of 20 clubs have gambling companies as front-of-shirt sponsors. ^5^ The increased prominence of gambling companies within sporting events has given rise to concern about the impact of these arrangements, especially on children and young people. ^6^ ^,^ ^7^

Some countries, such as Italy, Spain and the Netherlands, have implemented wide-ranging bans on sports sponsorship (as part of broader restrictions on gambling advertising) to curb gambling harms. ^8^ In Britain, a different approach has been adopted. In April 2023, the EPL announced that it would voluntarily ban front-of-shirt gambling sponsors from the 2026/27 season. ^9^ Two weeks later, the British Government’s White Paper, *High Stakes: Gambling reform for the digital age,* welcomed the EPL’s commitment to developing a voluntary cross-sport gambling sponsorship code rather than mandatory prohibitions. ^10^ The following year, in July 2024, the Premier League published its voluntary Code of Conduct for Gambling Related Agreements in Football, which applies to the EPL, the English Football League, the Football Association Challenge Cup (FA), and the Women’s Super League. The code was developed in consultation with the Department for Culture, Media and Sport, the Gambling Commission, the Betting and Gaming Council (an industry trade body), and the wider sports sector. The code enshrines four main principles for how Premier League teams should conduct their commercial gambling arrangements: protection of children and young people; social responsibility; reinvestment into sport; and maintaining sport integrity. ^11^ ^,^ ^12^ ^,^ ^13^ ^,^ ^14^

However, the most visible change proposed is the front of shirt sponsorship ban. This predominant focus overlooks a wider range of commercial arrangements between football teams and gambling companies. Specifically, commercial partnerships generate deeper, more integrated collaborations focused on mutual strategic and long-term value, often involving product development or tailored community initiatives. Sponsorships are a component of a partnership which focuses on brand visibility through advertising (e.g., logos on shirts). As the top teams in the EPL have partnered with, rather than been sponsored by, gambling companies, this demonstrates the deep integration of these commercial relationships. Moreover, an analysis of exposure to gambling advertisements in the EPL by Rossi et al. showed that gambling-sponsored front-of-shirt advertising accounted for just 9.66% of total marketing messages across the seven opening-weekend fixtures in the 2025/26 season. ^15^ As such, Rossi et al. argued that efforts to achieve substantial reductions in gambling exposure should account for the collective and ongoing nature of brand placement. This includes within stadia; regional advertising around the stadia and their locale; digital marketing; and partnership arrangements that target fans with exclusive competitions, prizes, experiences, betting odds and offers. ^16^ ^,^ ^17^ ^,^ ^18^

To date, a growing body of academic research has examined the historical development and nature of commercial partnerships between professional sports clubs and gambling companies. Some of the first studies mapped the rise in gambling front-of-shirt sponsorship, documenting the increasing visibility of gambling brands within professional football across the UK, and then globally. ^19^ ^,^ ^20^ ^,^ ^21^ ^,^ ^22^ Subsequent research by Torrance et al. demonstrated that gambling marketing extends far beyond shirt sponsorship, which accounted for only 6.9%; frequency analyses of EPL broadcasts identified extensive exposure to gambling and cryptocurrency branding through pitch-side advertising and other in-game promotional materials. ^23^ Ireland’s case study of one club showed that partnerships between gambling operators and football clubs increasingly rely on digital engagement techniques, including exclusive content, betting offers, and fan-focused marketing, to strengthen brand relationships with supporters. ^24^ More recently, De Jans et al.’s systematic review highlighted the need for a longitudinal, multi-analysis study on the diverse forms of sponsorship activation that extend beyond visible logo placement. ^25^ Understanding the types of arrangements that exist, how these partnerships have developed and varied over time, and how sports teams communicate these partnerships is important for understanding the depth of the commercial ties between these industries. Elucidating these patterns provides vital information for assessing the likely responses to the recent front-of-shirt sponsorship ban, and to examine the likely efficacy of the Premier League’s voluntary Code of Conduct.

To do this, this paper explores both the historic and recent commercial arrangements between five top-tier Premiership football clubs and commercial gambling companies from the 2000/01 to the present day (2025/26) to understand:

a) What kinds of commercial partnership arrangements exist with gambling companies over this time
b) To map trends in partnership arrangements across these football clubs
c) To highlight key risks and challenges in these commercial relationships
d) To examine the existing relationships which extend beyond front-of-shirt sponsorship and the extent to which they are integrated within the voluntary Code of Conduct principles.

The aim is to present the first descriptive data detailing historical commercial arrangements for five major EPL clubs, focusing on the number and types of commercial partnerships evident over this period.

## Methods

### Club selection

To explore the history, trends and depth of commercial gambling partnerships with the EPL, we examined the top five most successful Premier League clubs according to the Football365 website, which categorises clubs based on wins, draws, losses, goals for, goals against, and goal differences across our period of study from seasons 2000/01 to 2025/26. When filtering the table by these dates, from most to least successful, the following teams were selected: Manchester United, Arsenal, Chelsea, Liverpool, and Manchester City. ^26^ None of these clubs has ever had a front-of-shirt gambling sponsor, making them interesting case studies for exploring how commercial gambling partnerships develop in the absence of such an arrangement, and for charting the range and types of gambling partnerships and sponsorships.

### Partnership eligibility

Commercial partners were included if they offered any gambling or betting service to any population globally. Betting and gaming partners that operated in cryptocurrency were included, but the ‘cryptocurrency’ and ‘blockchain partners’ that focused on crypto trading, sales or which operated in more grey areas were not (i.e., Arsenal’s ‘cryptocurrency partnership’ with Cash Bet Coin in 2018).

### Search methods

To explore and document the wide range of commercial partnerships with gambling companies, we searched for partnership announcements published on either the football clubs’ or the gambling companies’ websites or within industry press, which sometimes lists sponsorship and marketing agreements. These searches were supplemented by examination of a range of digital archives (see Table 1).

**Table 1.** Search methods by coding theme.

Theme	Codes	Search method
Clothing	Front of shirt sponsorship	All clothing could be accurately searched for via the Football Kit Archive. Sleeve sponsorship was introduced in 2017/18 so coding for this is only applicable from this season onwards.
	Shirt sleeve/back of shirt sponsorship	
	Training kit	
Stadia	Stadia naming	Stadia naming was searched via club websites. Other forms of in-and-around stadia marketing were searchable on YouTube, or through online local and regional press. For each season, the logos of gambling companies on the hoardings for one to three home games were coded. As were press conferences and pre- or post-match interviews, held at each club’s stadium. From 2000/01 to early 2010s, the five teams transitioned from static to rotating/rolling and then digital LED perimeter hoardings. In 2017, new technology enabled hoarding advertisements to differ based on both the broadcaster and the region of digital viewership. We were only able to capture the hoardings advertisements shown in the UK. Hoarding advertisements shown outside the UK and kiosk branding were coded via partnership announcements only. Dedicated hospitality use was coded under other.
	Stadia kiosks	
	Advertising within stadia	
	Regional advertising around stadia	
	Media areas (press conference rooms; post-match interview spaces)	
	Other	
Digital integration	Integration on club digital assets, including official websites, fan hubs, social media, etc	Excluding Manchester City, searches focused on the ‘Partners and Sponsors’ pages of all the football clubs’ official UK websites and the club’s bespoke betting websites, every year since the early 2000s, via the Wayback Machine Internet Archive. The web page searches showed yearly co-branded content generation and social media posts, including access to the first team. Manchester City excluded both their first URL ( manchestercity.net) and most recent URL ( mancity.com) from being revisited; however, they did not exclude their in-between website, mcfc.co.uk, from 2006 to 2009. Nonetheless, once gambling partnerships were known, we instead searched the gambling companies’ websites of the 2010s via the Wayback Machine to locate digital integrations with the football club for Manchester City.
	Bespoke, club-branded gambling websites	
	Co-branded content generation, including access to first team	
Fan activations	Competitions/prizes and experiences	The two different types of deals, exclusive for the football clubs’ fans, were found via Wayback Machine, either on the football club or the gambling company websites, or via The British Newspaper Archives for local to regional press.
	Bespoke odds/gambling offers for fans	

### Coding

All textual reports were coded for the type of commercial arrangement offered. This identified 13 different types of commercial arrangements (see Table 1). This code frame was developed and piloted for Manchester City and then extended to all other football teams. We first coded responses based on textual data. Our website searches used the Wayback Machine and evidence from matchday videos on YouTube or fan websites to complete gaps in the code frame and verify information. Where data could not be verified, we used a ‘don’t know (DK)’ code, which has been excluded from the summary statistics presented. Finally, we checked if arrangements also applied to the women’s team.

Every team was double-coded by *FC* and *HW*, with inter-coder agreement of 90%. Minor discrepancies were discussed and resolved (mainly due to one coder finding an additional source of information, which allowed gaps in the code frame to be filled or additional evidence to be found around the start or end dates of contracts). Coding took place between November 2025 and April 2026. ^27^

### Missing data

For all clothing codes, the Football Kit Archive provided a complete record. For stadia codes, stadia naming is public information. The information on stadia kiosks was difficult to locate because they were not always included in the press announcements about the gambling partnerships. The media areas were also difficult to locate, as not all pre- or post-match interviews have been uploaded to YouTube ( Table 1). This data was coded by watching between one and three home games per season on YouTube. By combining YouTube game highlights with searches of local and regional press online, the code on regional advertising around stadia was more comprehensive. Except for stadium naming, the evidence for the stadium codes likely represents conservative estimates.

For all digital integration codes, the Wayback Machine (Internet Archive) served as a near-comprehensive record for all clubs, except when searching Manchester City’s website outside of the years 2006 to 2009 ( Table 1). The fan activation codes were also near-comprehensive for all clubs via the Wayback Machine, the gambling companies’ websites, or The British Newspaper Archives.

### Temporal trends

To examine changes over time across all clubs, data were grouped into five-year bands to examine changes in the quantity and nature of marketing across this period (see Figure 2). Commercial deals were allocated to a year group based on whether the partnership commenced in that year. As the specific end date of each partnership was not always specified, this analysis examines trends in marketing features for partnerships commencing within each five-year band, regardless of duration. Some partnerships will have spanned multiple years; thus, this analysis does not represent the total number of partnerships held within each period, but rather the total number of new partnerships commenced. The first group covered the seasons 2000/01 to 2004/05; then the next covered 2005/06 to 2009/10, and so on. Whilst caution is warranted when comparing some categories over time (such as stadia kiosks, whose presence was more difficult to verify), most other categories, particularly fan activations, have robust, verified data from the Wayback Archive and other online resources.

## Results

### Characteristics of commercial arrangements

Overall, we identified 91 distinct gambling partnerships across all five clubs between 2000/01 and 2025/26 ( Table 2). Manchester City F.C. had 21 gambling partnerships dating back to at least 2002/2003, including an early deal with Ladbrokes, which featured advertising on pitch-side hoardings, integration with the club’s digital assets, exclusive fan odds/competitions, and access to the first team for cross-promotions. This was followed by Chelsea F.C., with 20 commercial partnerships, including the government-backed venture TotalBet (in a relationship that predates our search period), with a deal that included exclusive competitions and odds/offers, first-team access, and digital integration with club assets. Liverpool F.C. also had 20 distinct commercial gambling partnerships, dating back to at least 2000/01, including an early deal with Eurobet (a precursor of Coral’s online website) for pitch-side hoardings. Arsenal had 16 commercial gambling partnerships, commencing with Ladbrokes at the very least in 2002/03, which included pitch-side hoarding adverts, logo display during interviews, integration into the club’s digital assets, first-team access for promotions, exclusive competitions and odds/offers. Finally, Manchester United had 14 commercial gambling partners, starting with Coral/Eurobet, at least since 2000/01, which included stadium kiosks, pitch-side hoardings, integration into digital assets, exclusive competitions and odds/offers, access to the first team, and a bespoke Manchester United Official betting website.

**Table 2.** The numbers and percentages of thirteen types of sponsorship, under four themes, for each club. *

Club	Total number	Clothing			Stadia					Digital integration			Fan activation	
		Front of shirt	Sleeve	Training kit	Stadia naming	Stadia kiosks	Advertising within stadia	Regional advertising around stadia	Media areas, etc	Club digital assets	Co-branded content	Bespoke gambling website	Competitions, prizes, experiences etc	Exclusive odds, betting offers
		N (%)	N (%)	N (%)	N (%)	N (%)	N (%)	N (%)	N (%)	N (%)	N (%)	N (%)	N (%)	N (%)
Man U	14	0 (0.0%)	0 (0.0%)	0 (0.0%)	0 (0.0%)	3 (21.4%)	10 (71.4%)	7 (50%)	4 (28.6%)	12 (85.7%)	14 (100.0%)	5 (35.7%)	13 (92.9%)	13 (92.9%)
Man C	21	0 (0.0%)	0 (0.0%)	2 (9.5%)	0 (0.0%)	2 (9.5%)	18 (85.7)	17 (81%)	4 (19%)	11 (52.4%)	18 (85.7%)	1 (4.8%)	18 (85.7%)	18 (85.7%)
Chelsea	20	0 (0.0%)	0 (0.0%)	0 (0.0%)	0 (0.0%)	2(10%)	9 (45%)	13 (65%)	1 (5%)	16 (80%)	17 (85%)	1 (5%)	15 (75%)	17 (85%)
Arsenal	16	0 (0.0%)	0 (0.0%)	0 (0.0%)	0 (0.0%)	2 (12.5%)	14 (87.5%)	16 (100%)	2 (12.5%)	16 (100%)	14 (87.5%)	1 (6.3%)	16 (100%)	17 (106.3%)
Liverpool	20	0 (0.0%)	0 (0.0%)	1 (5.0%)	0 (0.0%)	0 (0.0%)	14 (70%)	10 (50.0%)	1 (5.0%)	11 (55.0%)	12 (60.0%)	0 (0.0%)	12 (60%)	12 (60%)
**Total**	**91**	**0 (0.0%)**	**0 (0.0%)**	**3 (3.3%)**	**0 (0.0%)**	**9 (9.9%)**	**65 (71.4%)**	**63 (69.2%)**	**12 (13.2%)**	**66 (72.5%)**	**75 (82.4%)**	**8 (8.8%)**	**74 (81.3%)**	**77 (84.6%)**

* The thirteen codes of commercial arrangements are not mutually exclusive; as a single partnership can span more than one category, the percentages are not intended to sum to 100%.

The nature of these partnerships varied, with some classified as regional partners and others as global betting partners. Multiple gambling partners often overlapped. For example, Manchester City F.C. had four different gambling partners for the 2005/06 season. This trend was more common in the mid-2010s, when technological advances enabled greater tailoring to regional markets and allowed pitch-side hoardings to display different companies based on the viewer’s location. In 2016 alone, Arsenal F.C. had nine regional gambling partners.

Typically, the types of gambling promoted by companies with whom clubs had commercial arrangements focused primarily on betting, and the arrangements were often described as official betting partnerships. Across all 91 partnerships identified, 77 (84.6%) included evidence of the gambling partner offering preferential betting odds to fans. Some partnerships were for casino, slots, or arcade products, such as Manchester United F.C.’s partnership with overseas casinos KamaGames and Donaco. Except for Liverpool F.C., all clubs have increasingly partnered with cryptocurrency-based betting or casino companies. In 2024, Chelsea simultaneously partnered with three (Roobet, TMGM, and BingX).

***Specific commercial arrangements***

Specific commercial arrangements for each club are shown in Figure 1 (front-of-shirt sponsorship and stadium names were omitted because none of the commercial partnerships included these).

**Figure 1.**
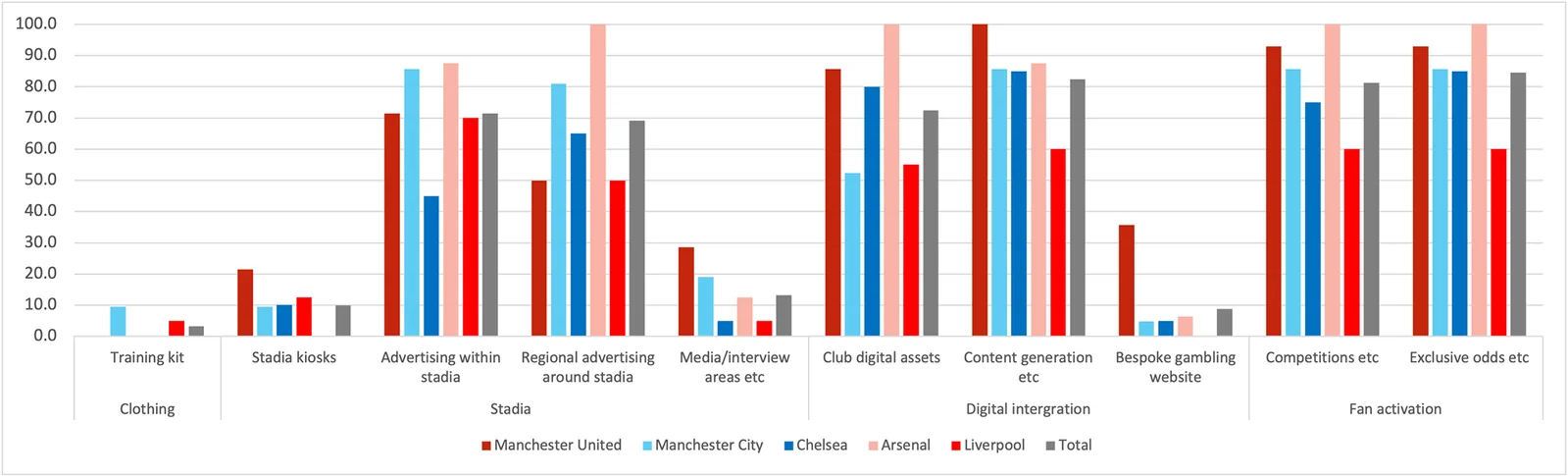
The numbers and percentages of thirteen types of sponsorship, under four themes, for each club.

***Clothing sponsorship (front-of-shirt, sleeve, and training kit)***

None of the five clubs had a front-of-shirt or shirt-sleeve gambling sponsor (permitted from 2017/18) between 2000/01 and 2025/26. Sponsorship of the training kit varied across clubs. Between 2016/17 and 2018/19, Liverpool’s official training kit sponsor was BetVictor. From 2019/20 to 2020/21, Manchester City’s training kit was sponsored by Marathon Bet, and in 2022/23, LeoVegas was the sponsor on the sleeve of their training kit.

***Stadia sponsorship (stadium naming, in-stadia advertising, regional advertising around stadia, interview areas, kiosks)***

Across all clubs, stadium naming did not feature; however, in-stadia advertising via pitchside hoardings was common. Overall, over two-thirds of commercial partnerships identified included brand advertising on pitch-side hoardings (71.4% of the commercial deals analysed included this). Regional advertising in and around the stadium accounted for over two-thirds of the commercial deals coded (69.2%). Brand logo placement in media interview areas was present in 13.2% of deals, and 9.9% included stadia kiosks.

***Digital integration (official club website, fan hubs and social media, bespoke club-branding gambling websites, and co-branded content generation)***

Overall, digital integration with club assets was a very common feature of these commercial arrangements, appearing in 72.5% of coded partnerships. Co-creation of content for digital assets, including co-branding and access to the first team for promotion, was also very common, present in 82.4% of coded partnerships. Interestingly, all clubs except Liverpool established at least one of their own bespoke gambling website, with Manchester United developing five in total. Since 2019, all five clubs have instead promoted their partners’ websites rather than building their own bespoke gambling infrastructure.

***Fan activations (competitions, prizes and experiences, and bespoke odds/offers)***

Fan activations were the predominant feature of most commercial partnerships, offering exclusive odds or experiences to fans, offered both online and within stadia. Offering exclusive betting odds to fans was a feature in 84.6% of coded partnerships, with bespoke competitions and prizes hosted by the gambling companies also offered in 81.3% of partnerships. Although odd boosts were the most common, other examples included early pay-outs (Betfred and Manchester United), VIP experiences to meet the players (Betway and Chelsea), and hospitality suites which provided premium matchday experiences (Betfair and Arsenal).

***Women’s teams***

Over the period under consideration, these commercial arrangements did not typically extend to women’s teams, likely reflecting the more recent commercialisation of women’s football and historically rooted gender preferences that have led to gambling being marketed primarily to men. ^28^ However, for both Liverpool F.C. and Manchester City F.C., the deals are increasingly including the women’s teams. For example, from 2016/17, BetVictor’s sponsorship of Liverpool’s training kit included the women’s team, and Manchester City F.C.’s 2022/23 regional partnership with LeoVegas included the women’s team.

***Changes over time***

From our analysis, the total number of new commercial partners across all five clubs increased from 16 over the five-year period from 2000/01 to 2004/05 to 21 over the five-year period from 2019/20 to 2025/26. (see Table 3). The nature of the marketing features included within these deals also changed over time. Overall, there was an increase in the number of commercial deals including fan activations: between 2000/01 and 2004/05, only 50 to 62.5 per cent of commercial deals included fan activations; this increased to over 90 per cent for the period 2019/20 to 2025/26 (see Figure 2). Furthermore, there was evidence that commercial deals included a wider range of features in the later period, with over 90% of commercial deals commencing from 2019/20 onwards including at least three different marketing techniques *.* Interestingly, there was little evidence of a substantial increase in the proportion of deals which included in-stadia advertising. This varied from 87.5% in commercial deals commencing between 2000/01 and 2004/05, to 76.2% for deals starting from 2019/20 onwards. This is despite an increase in the overall number of commercial deals observed across this period (see Figure 2).

**Table 3.** The percentage of new commercial deals containing selected marketing features, by five-year intervals from 2000/01 to 2025/26.

	2000/01 to 2004/05	2005/06 to 2009/10	2010/11 to 2014/15	2015/16 to 2019/20	2020/21 to 2025/26
Stadia: Advertising within stadia (hoardings etc)	87.5	78.9	33.3	60.9	76.2
Stadia: Regional advertising around stadium	50.0	73.7	75.0	65.2	66.7
Logo display - interview areas	12.5	15.8	16.7	13.0	9.5
Digital integration: Club/fan websites etc	56.3	73.7	66.7	82.6	66.7
Digital Integration: Co-branded content/social media	62.5	68.4	91.7	78.3	95.2
Fan Activations: Competitions, prizes or experiences	50.0	78.9	83.3	78.3	95.2
Fan Activations: Exclusive odds, offers	62.5	78.9	91.7	82.6	90.5

**Figure 2.**
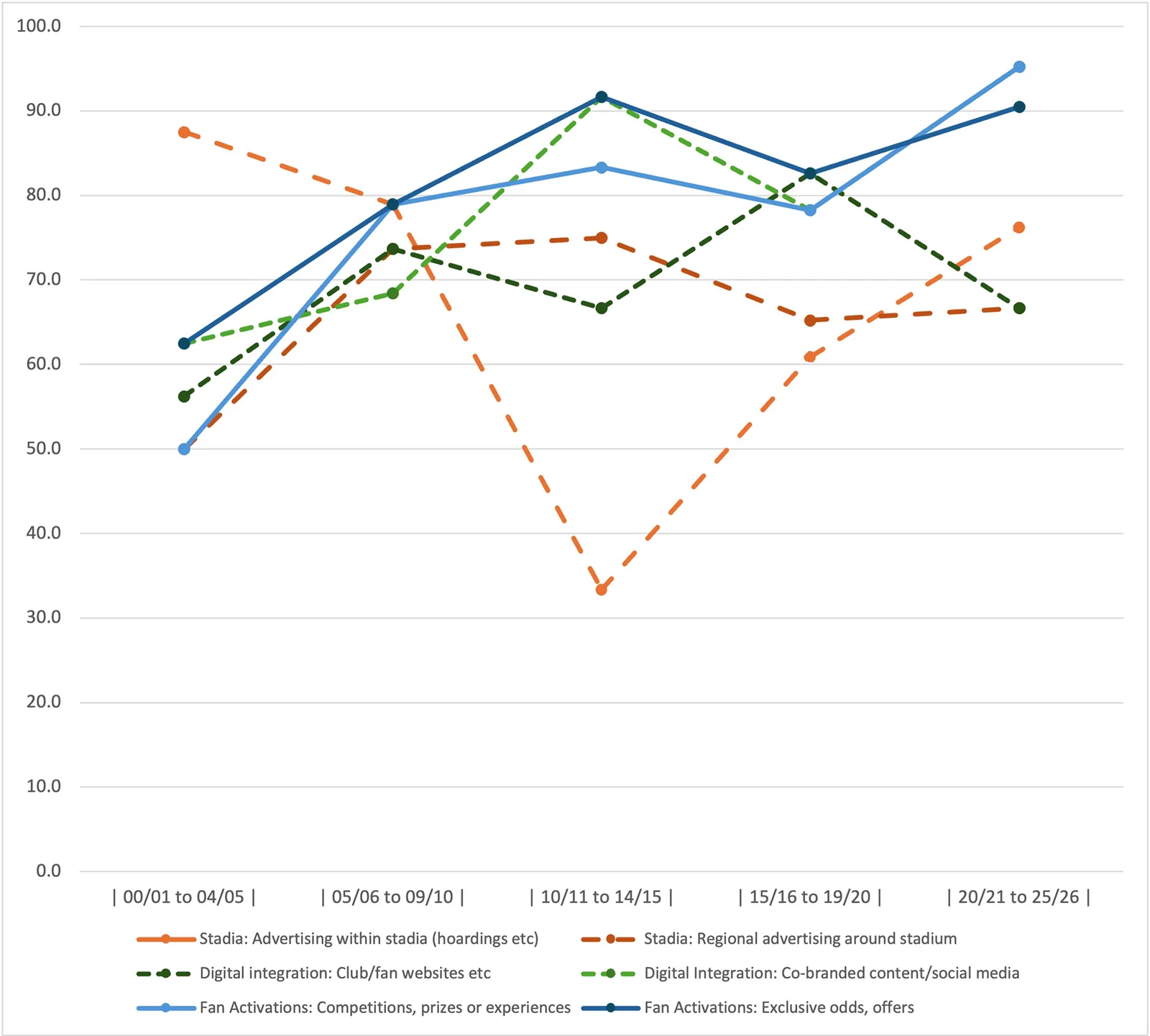
The percentage of new commercial deals containing selected marketing features, by five-year intervals from 2000/01 to 2025/26. The percentage of new commercial deals containing selected marketing features, by five-year intervals from 2000/01 to 2025/26.

## Discussion

Over a 25-year period, our analysis identified at least 91 different commercial partnerships between gambling companies and the five leading EPL football clubs. These partnerships were established before the start of our study period (2000/01), reflecting the presence of non-shirt gambling sponsorship in the 1990s as part of the EPL’s longer history with gambling sponsorship since its inception. ^29^ The partnerships included a wide range of arrangements, such as in-and-around-stadium advertising, integration with digital assets, co-production and cross-promotion utilising the first-team, and specific and targeted fan activations. Over time, the number of these relationships has increased, with some clubs entering multiple arrangements with different firms in the same season. The expansion was facilitated by the government’s relaxation of legislation in the early 2000s that had prohibited public gambling marketing. This led to a rise in simultaneous partnerships with domestic companies. The expansion was further intensified by the rise in region-specific partnerships with overseas gambling firms following advances in digital marketing technologies, such as the capacity—introduced in the mid-2010s—to display different pitch-side branding to audiences in different geographic locations.

Notably, none of the five clubs had front-of-shirt sponsors but maintained deep, reciprocal commercial gambling partnerships with a wide range of companies, specifically granting partners access to several activations and activities that encourage gambling. Fan activations were a more prominent feature within these commercial deals than in-stadia advertising. In the marketing literature, fan activation is described as the process of turning fans from passive recipients into active participants. In this case, active participants in gambling. ^30^ The locations of these activations expanded from printed ephemera and websites to texts and emails, and then to apps, through which fans are reached more directly, regularly, and discreetly.

Our temporal analysis also shows that the number of commercial deals with different partners increased over the 25-year period, as did fan activations and digital integration, particularly through co-branded content generation, which was a key feature of these deals. There is limited evidence of increased breadth in-stadia advertising, suggesting a likely saturation point in how many different brands can be supported to advertise specifically within one stadium (and the associated cost of doing so). Instead, fans are increasingly targeted by a wider range of gambling partnerships that aim to engage them as active gamblers through fan activations and digital integrations. This is likely enabled by digital segmentation across international markets.

Research evidence shows these kinds of activities have an impact; around one-third of people who gamble stated that exposure to advertising and marketing activity encouraged them to gamble when they did not intend to do so, with 87% of those experiencing problem gambling reporting the same. ^31^ Equally, research evidence shows a dose-response relationship between increased advertising/marketing and gambling consumption, whereby increased gambling consumption (especially beyond certain thresholds) is associated with a higher risk of harm. ^32^ ^,^ ^33^ In addition, content generation, a key feature of digital integration embedded in commercial practice, is described as “as the conduit for brands to reach their intended audience, enhancing the customer relationship through access to their heroes”. ^34^

### Marketing trends and integration with voluntary codes of practice

Our evidence shows an increased presence of a wide range of commercial marketing practices beyond front-of-shirt sponsorship. Notably, none of these increasing practices was subject to specific focus within the Premier League’s Voluntary Code of Conduct for Gambling Related Agreements in Football, co-developed with the gambling industry.

For example, the code enshrines a principle to ‘protect children and other vulnerable persons’, defined as ‘those at risk of gambling-related harm’, which applies only to those aged under 18, and its recommendations cover only replica kits or children’s sections of websites. Although the logos of gambling sponsors have been removed from all five official clubs’ websites at the beginning of the 2026/27 season, excluding their partnership webpages (August 2026), children will continue to be exposed to any fan activations advertised in and around stadia, as well as through co-created content within the club’s social media channels. Even if major social media platforms are banned for under-16 s by Spring 2027 in the UK, children over this age will remain exposed to this form of gambling marketing. ^35^ ^,^ ^36^

The code’s section on social responsibility focuses on responsible gambling messaging and the provision of workshops to raise awareness among professional footballers about gambling disorder. However, this commitment to social responsibility is potentially undermined by the expansion of fan activations. The code does not address the rules or regulations governing exclusive odds, offers, experiences or competitions to promote gambling companies, other than requiring that such promotions be accompanied by responsible gambling messaging. Since October 2022, current professional players, including the first team of each club, cannot be used in UK gambling marketing, ^37^ including sponsorship, but they can feature in other countries, depending on each country’s jurisdiction. Yet, as soon as players retire, gambling partners can and do deploy prestige players from these top clubs to feature in gambling marketing. Whilst there is a requirement to include appropriate responsible gambling messaging across gambling marketing, research by Newall has shown that some messages have little positive impact and, in some cases, may be counterproductive. ^38^

Finally, the actions taken since the announcement of the front-of-shirt ban show the myriad ways these clubs and gambling corporations are adapting to the policy. Gambling logos remain permissible on training kits, shirt sleeves, stadia, and, as we have shown, within digital and fan activation marketing. In response to the front-of-shirt ban, Manchester United have arranged training kit and shirt-sleeve sponsorships with Betway for the 2026/27 season for both the men’s and women’s teams. In addition, cryptocurrency sponsorship, which operates in a grey area regarding gambling, ^39^ ^,^ ^40^ is increasing in its clothing sponsorship. For the 2026/27 season, Manchester City’s men’s training kits are sponsored by OKX, and Chelsea’s men’s and women’s training kits by BingX. The commercial success of the women’s team has created new marketing avenues for gambling, cryptocurrency, and trading partnerships.

### Limitations

There are several limitations with this paper. First, it was difficult to locate full information on each commercial arrangement. The estimates quoted here for each team are likely conservative due to the lack of transparency about exactly what each partnership entails. Our coding for clothing was the easiest to find because of its high visibility, which may explain why it is a key focus in the Voluntary Code. Excluding Manchester City F. C, the information on the digital integration and fan activation was also relatively straightforward to document using digital archives. However, aside from naming rights, the information on the commercial deals for all forms of stadium advertising, particularly kiosks, lacked significant transparency; these figures are likely higher.

Despite this, we are confident that the observed growth in gambling partnerships is not due to a lack of information on older partnerships; the Wayback Machine (Internet Archive) was highly reliable for coding past partnerships and their features, particularly for digital integration and fan activations. That said, it did become harder to locate more detailed information on partnership arrangements from the mid 2010s because club press releases either did not occur (i.e., particularly when the new gambling partners operated overseas) or stopped listing all the sponsorship arrangements (e.g., stadia kiosks), or the club requested the exclusion of their online records (i.e., Manchester City’s club website on the Wayback Machine (Internet Archives)). There may have been additional partners, or partnership features that we were unable to locate in this period.

Second, we considered only the five most successful premiership clubs, which have global reach and arguably different economic models from those of mid- or lower-tier clubs. Thus, the results are specific to these clubs and are not necessarily generalisable to all clubs. It would be interesting to repeat the exercise with lower-league clubs. However, the five clubs chosen did not have front-of-shirt sponsorship arrangements and thus represent a potentially interesting case study of how sports teams and football clubs interact in the absence of visible shirt sponsorship.

Third, although these clubs did not have front-of-shirt sponsorship for EPL games, they have worn shirt sponsorship from gambling operators in other football competitions. Whilst not an official EPL club partnership, it’s notable that seniors from three clubs wore Empire Poker shirts (Chelsea, Liverpool and Manchester City) in 2005, as the gambling firm was that year’s official partner for the Masters Football competition (2000–2011, 2022-present). In 2025, the gambling firm Play OJO became the competition’s official title and shirt sponsor. In 2026, Play OJO was replaced by Ladbrokes, highlighting the myriad ways in which commercial gambling companies foster relationships with football and, indirectly, with clubs through promotions featuring former club legends.

## Conclusion

To conclude, Premier League football clubs maintain deep, reciprocal relationships with gambling companies that are designed to influence fan behaviour—even in the absence of front-of-shirt sponsorships. Targeted fan activations that actively encourage betting are as widespread as traditional forms of advertising, such as pitchside hoardings, and appear to be increasing. Crucially, these practices fall outside the scope of the Premier League’s voluntary Code of Conduct for Gambling-Related Agreements in Football. Whilst the ban on front-of-shirt sponsorship may reduce the visibility of gambling logos, other forms of exposure are likely to persist through a wide range of marketing strategies that remain largely unregulated and lack effective oversight or governance.

Finally, the considerable effort required to identify, verify and document these partnerships highlights a broader lack of transparency surrounding football clubs’ commercial relationships, making it difficult for researchers, policymakers and the public to fully understand the scale, nature and changes in gambling-related marketing practices. This lack of transparency has important policy implications, as regulators and policymakers cannot effectively assess, monitor or govern gambling-related commercial practices when the scope and content of partnership agreements are not routinely disclosed.

## Data Availability

Data are available under the terms of the Creative Commons Attribution 4.0 International license (CC-BY 4.0): https://doi.org/10.5281/zenodo.22079561: Creed F, Wardle H. Dataset of Gambling Partnerships and 13 Types of Sponsorship across 5 English Premier League Clubs (2000/01–2025/26). Zenodo [Data set]. 2026.
