# Supplementary material for: Behind the Shirt: Examining Trends in the Commercial Relationships Between English Premier League Football Clubs and Gambling Companies
Source: Wellcome Open Res. 2026 Oct 6;11:813. [Version 1] doi: 10.12688/wellcomeopenres.27602.1 (PMC13728439; doi:10.12688/wellcomeopenres.27602.1)
Supplement: Peer review history

# Reviewer report: Reviewer response for version 1

**DOI:** 10.21956/wellcomeopenres.30385.r173224

**Published:** 2026-11-02

**Authors:** Jennifer T. Grant Weinandy

**Affiliations:** 1 Ohio University, Ohio, USA

This study assesses relationships between gambling companies and five top Premier League Teams since 2000. It focuses on the breadth of ways in which gambling companies may partner with teams and, thus, promote gambling to fans outside of “front-of-shirt” logos (which have recently been banned). This work also notes how such partnerships have changed over time, seemingly increasing and diversifying. In this way, this study highlights an important gap in current regulation, in that the ban on “front-of-shirt” logos likely does not attend to the true nature of such partnership or potentially effectively reduce the harm it intends to.

 I had a couple of thoughts about ways to improve the manuscript, primarily in terms of clarifying some of the methods:

**Method:**

1. I think focusing on the statistics and who is considered most successful seems reasonable when selecting teams; however, it is unclear why 5 was the number of teams chosen. Was this just considered likely to be a good representation? Clarity about your reasoning here should be presented.
2. It seems like you did not necessarily pick teams based on whether they previously had front-of-shirt sponsorship, but this should be clarified. If you did pick this deliberately, would there be a benefit to comparing to teams with front of shirt sponsorship at some point?
3. Did the code frame change at all after extending it to teams outside of Manchester United? Or did you maintain the code frame and only look for those partnerships? Some clarity on how the coding developed here is needed here.

**Results:**

1. Figure 1 appears to present just percentages, but is named “numbers and percentages,” I suggest changing it to just percentages.
2. You note that many of these clubs had their own bespoke gambling infrastructure, was there any advertising for this that you could code for? I wonder if this would change their partnership decisions, since it is possible other gambling partners pre-2019 could have been seen more as competition for their own infrastructure, but now that competition is gone leading to increased partnerships.

**Discussion:**

1. There appears to be a little dip in stadia advertising from 2010-2015, are there any regulation changes or factors that may have caused this? Is it due to the poor transparency of some agreements around this time that made it hard to map?
2. Given that some of the partnerships that are discussed relate to relationships with the team (e.g., on kits) and their involvement in advertisements, I wonder whether highlighting this particular concern may be relevant. I am particularly thinking about work by D’Ambrogio et al. (2023) showing that celebrity status in advertisements can bias decision making.

Thank you for the opportunity to review this work.

Is the work clearly and accurately presented and does it cite the current literature?

Yes

If applicable, is the statistical analysis and its interpretation appropriate?

Not applicable

Are all the source data underlying the results available to ensure full reproducibility?

Yes

Is the study design appropriate and is the work technically sound?

Yes

Are the conclusions drawn adequately supported by the results?

Yes

Are sufficient details of methods and analysis provided to allow replication by others?

Partly

Reviewer Expertise:

Behavioral addictions and Gambling Disorder, including gambling harm, access to care, and harm minimization efforts

I confirm that I have read this submission and believe that I have an appropriate level of expertise to confirm that it is of an acceptable scientific standard, however I have significant reservations, as outlined above.

## References

[1] : How celebrity status and gaze direction in ads drive visual attention to shape consumer decisions. *Psychology & Marketing* .2023;40(4) : 10.1002/mar.21772723-734 10.1002/mar.21772

# Reviewer report: Reviewer response for version 1

**DOI:** 10.21956/wellcomeopenres.30385.r173220

**Published:** 2026-10-24

**Authors:** Steffi De Jans

**Affiliations:** 1 Ghent University, Ghent, Belgium

This manuscript investigates the commercial gambling partnerships with five top-performing EPL clubs from season 2000/2001 to 2025/2026. The research addresses a timely and relevant topic, and the manuscript is generally well-written. However, several areas would benefit from further clarification and elaboration.

 Abstract: Which clubs were investigated? The Background section refers to leading EPL clubs that have never had front-of-shirt gambling sponsorships, whereas the Methods section refers to the five top-performing EPL clubs. This becomes clear in the article, but could be made more clear in the abstract as well.

 In the first paragraph of the Introduction, the statement that partnerships and sponsorship “have increased over the last 25 years” does not make clear which context is meant. Does this refer to the situation worldwide or in the UK? I would recommend adding context here.

 Please formulate the main research aim consistently. At present it is stated both before and after the four research questions are presented.

 In the section on search methods, the authors should describe the search of digital archives in more detail. Could they explain how in-stadium and around-stadium marketing could be searched on YouTube?

 The coding procedure could be explained in somewhat more detail. How did the authors arrive at the four central themes, and why is fan activation treated separately from digital integration, given that fan activation will often also take place digitally? Fan activation is defined in the Discussion, but this explanation should be provided earlier to help the reader. In addition, the codes within digital integration should be explained in more detail, as it is not clear to me what each code entails. For example, what exactly is meant by club-branded gambling websites? The same applies to all results on digital integration (e.g., what exactly does co-branded content generation involve?). Please provide descriptions and possibly examples to make this clearer. I think that all themes and codes need to be defined more concretely and grounded in the literature.

 The coding scheme was developed on the basis of the Manchester City case. If other clubs had different arrangements, could these still be added to the scheme? Information is also needed on how the website searches were conducted.

 The inclusion of both men’s and women’s teams should be explicitly mentioned in the club selection section.

 What is meant by first-team access?

 Discussion: What is meant by the statement that the partnerships were established before the study period?

 Could the authors explain the “reciprocal” aspect of the commercial gambling partnerships?

 The authors state that fan activation was present in, for instance, emails, text messages and apps. How was this covered by the search?

 The paragraph on social responsibility in the Discussion does not entirely align with what this study has shown. For instance, the study did not take responsible gambling messages into account. That is acceptable, but it is then somewhat odd to end the paragraph with a statement that such messages have little effect.

 Thank you for the opportunity to review this manuscript. I hope these comments are helpful to the authors, and I wish them the best with their research.

Is the work clearly and accurately presented and does it cite the current literature?

Yes

If applicable, is the statistical analysis and its interpretation appropriate?

Yes

Are all the source data underlying the results available to ensure full reproducibility?

Partly

Is the study design appropriate and is the work technically sound?

Yes

Are the conclusions drawn adequately supported by the results?

Yes

Are sufficient details of methods and analysis provided to allow replication by others?

Partly

Reviewer Expertise:

Gambling advertising

I confirm that I have read this submission and believe that I have an appropriate level of expertise to confirm that it is of an acceptable scientific standard, however I have significant reservations, as outlined above.

# Reviewer report: Reviewer response for version 1

**DOI:** 10.21956/wellcomeopenres.30385.r173223

**Published:** 2026-10-24

**Authors:** Filip Kovařík

**Affiliations:** 1 Palacký University Olomouc, Olomouc, Czech Republic

I am pleased to have been given the opportunity to review this paper. To my best knowledge this is the first study of its kind which I consider a strength. It is timely, has clear political application, and works rigorously with the dataset. I personally find the focus on „invisible“ forms of marketing (like fan activations or digital integration) very interesting and welcome it. The distinction between partnership and sponsorship is useful as I feel it is considered the same thing sometimes.

 The choice of clubs is reasonable and clearly explained, and I applaud the focus on the „larger“ clubs, as these major brands do not typically feature gambling sponsors on their shirts, securing more lucrative deals with „mega-companies“ instead (like airlines or finance sector). The coding process is sound. With this kind of research it is understandable that there would be some data missing or it would not be possible to check all of it.

 I want to raise just a few recommendations:

- **Intro**: You cite the share of front-of-shirt advertis from Rossi (9,66 %) and Torrance (6,9 %). For Torrance, the basis of the figure is not stated alongside the number. I would recommend briefly stating what each figure is calculated from, so that they are not read as directly comparable.
- **Methods**: I understand that the clubs were selected on the success basic. However in the EPL context the so-called „Big six“ (including Tottenham) seems a natural choice, particularly in terms of global reach and commercial strength. Why was success based selection preferred to the Big six?
- **Discussion**: In my view, the conclusion regarding a „saturation point“ is not fully supported by the data. The indicator analysed (share of new deals including pitch-side advertising) says nothing about the number of brands active concurrently in a single stadium. I would suggest that the stable share can be explained by the fact that pitch-side advertising is a standard component of the package (mostly owing to TV rights). The argument about limited capacity is somewhat at odds with your finding on regional display on a single board. I would suggest either counting concurrently active pitch-side partners per club per season, or rewording the explanation as a „possible explanation“.
- **Conclusion:** The finding on lack of transparency is interesting, but I suggest adding a concrete recommendation here. For example - should the code be extended to require disclosure of the content of partnership agreements.

Further comments:

- Clubs wear different shirts in European competitions (UCL, UEL, UECL) compared to their domestic league sometimes with these sponsorships often included in marketing contracts. I’m aware the paper focuses on the EPL but it would be interesting to know whether you encountered any such cases.
- I believe the references 4 + 20 (Bunn); 16 + 24 (Ireland) and 30 + 34 (Seymour) are duplicates.

Is the work clearly and accurately presented and does it cite the current literature?

Yes

If applicable, is the statistical analysis and its interpretation appropriate?

Not applicable

Are all the source data underlying the results available to ensure full reproducibility?

Yes

Is the study design appropriate and is the work technically sound?

Yes

Are the conclusions drawn adequately supported by the results?

Yes

Are sufficient details of methods and analysis provided to allow replication by others?

Yes

Reviewer Expertise:

Gambling; sports; advertising

I confirm that I have read this submission and believe that I have an appropriate level of expertise to confirm that it is of an acceptable scientific standard.
